# Mutation Profiles, Glycosylation Site Distribution and Codon Usage Bias of Human Papillomavirus Type 16

**DOI:** 10.3390/v13071281

**Published:** 2021-06-30

**Authors:** Wei Liu, Junhua Li, Hongli Du, Zhihua Ou

**Affiliations:** 1 School of Biology and Biological Engineering, South China University of Technology, Guangzhou 510000, China; weiliu_scut@163.com (W.L.); lijunhua@genomics.cn (J.L.); hldu@scut.edu.cn (H.D.); 2 BGI-Shenzhen, Shenzhen 518083, China; 3 Shenzhen Key Laboratory of Unknown Pathogen Identification, BGI-Shenzhen, Shenzhen 518083, China

**Keywords:** HPV16, lineage and sublineage, mutation, glycosylation, codon usage bias

## Abstract

Human papillomavirus type 16 (HPV16) is the most prevalent HPV type causing cervical cancers. Herein, using 1597 full genomes, we systemically investigated the mutation profiles, surface protein glycosylation sites and the codon usage bias (CUB) of HPV16 from different lineages and sublineages. Multiple lineage- or sublineage-conserved mutation sites were identified. Glycosylation analysis showed that HPV16 lineage D contained the highest number of different glycosylation sites from lineage A in both L1 and L2 capsid proteins, which might lead to their antigenic distances between the two lineages. CUB analysis showed that the HPV16 open reading frames (ORFs) preferred codons ending with A/T. The CUB of HPV16 ORFs was mainly affected by natural selection except for E1, E5 and L2. HPV16 only shared some of the preferred codons with humans, which might help reduce competition in translational resources. These findings increase our understanding of the heterogeneity between HPV16 lineages and sublineages, and the adaptation mechanism of HPV in human cells. In summary, this study might facilitate HPV classification and improve vaccine development and application.

## 1. Introduction

Human papillomaviruses (HPVs) cause mucosal and cutaneous infections. Up to now, more than 200 different HPV types have been identified (https://www.hpvcenter.se/human_reference_clones/ accessed on 30 November 2020). According to their carcinogenicity, HPVs can be divided into high-risk and low-risk types. High-risk types include HPV16, 18, 31, 33, 34, 35, 39, 45, 51, 52, 56, 58, 59, 66, 68 and 70 [1], which can cause cervical cancer. Among them, HPV16 is the dominant type and accounts for above 50% of cervical cancer cases [2,3].

HPVs are double-stranded circular DNA viruses with a genome size of about 8kb. HPV16 genomes include three general regions: a region encoding early-stage proteins (E1, E2, E4, E5, E6 and E7), a region encoding late-stage proteins including L1 and L2, and an upstream regulatory region (URR) [4]. E1 and E2 proteins regulate the replication and transcription of HPV genomes [5,6]. E4 overlaps with the E2 ORF and its product plays a role in genome amplification and virus synthesis [7]. E5, E6 and E7 proteins are cofactors for HPV carcinogenesis, and are involved in epithelial dysplasia and tumor progression after HPV infection [7,8,9,10]. L1 and L2 are the major and minor capsid proteins, which are expressed during the late stage of HPV infection. Besides forming the elegant icosahedral surface of the papillomavirus virion, these two capsid proteins are essential for virus binding and entry into cells [11,12]. Currently, L1 and L2 proteins, especially L1, are the component of HPV prophylactic vaccines [13], while E6 and E7 are part of the therapeutic vaccines of HPV-induced lesions and cancers [14].

Above the type level, HPVs are classified based on the nucleotide sequence of L1 [15,16]. In 2013, Chen et al. proposed the lineage/sublineage classification criteria for papillomaviruses of the same type based on the nucleotide difference of complete genomes, with 1.0–10.0% and 0.5–1.0% differences defining different lineages and sublineages [17]. Up to date, four lineages (A–D) and sixteen sublineages (A1–4, B1–4, C1–4 and D1–4) have been identified for HPV16 around the world [18,19]. Unfortunately, full viral genomes were not easily available in clinical settings or large-scale epidemiological studies. Therefore, specific mutations in partial genomic regions have been used for the classification of lineages or sublineages [20]. It would be informative to explore the lineage/sublineage-related polymorphisms using an updated dataset of complete genomes, which may reveal new marker sites with higher specificity.

It has been reported that HPV16 sublineages differ in their geographic distribution and carcinogenicity [21,22,23]. Sublineage A1 was the dominant sublineage in Europe, the Americas, South Asia and Oceania, and sublineage A2 was distributed in Europe, North America and Oceania, while sublineages A3 and A4 were mainly distributed in East Asia. Lineage B and C were almost exclusively distributed in Africa, and lineage D was the most common in South/Central America [24]. Mirabello et al. found that white women infected with HPV16 A1/A2 variants had an increased risk of CIN3+ (cervical intraepithelial neoplasia grade III) progression, and sublineage A4 was associated with an increased risk of cancer in Asian women [25]. A better understanding of the lineage/sublineage conserved mutations could facilitate the large-scale correlation study on the carcinogenicity of HPV16 variants. 

Glycosylation, especially occurring in viral surface proteins, may interfere with the antigenicity of viruses, which is related to vaccine development. N-linked glycosylations are mainly observed in the N-X-T/S (X: any amino acid except for P) motifs. Nucleotide mutations leading to the gain or loss of such motifs would modify the number of glycosylation sites in the proteins, affecting the binding affinity between viral proteins and the cellular receptors or antibodies. For example, mutation in the N-glycosylation motif of the surface envelope glycoprotein of HIV, gp120, could remove the glycosylated oligosaccharide chain and expose the masked antigenic epitopes, increasing the antigenic recognition of gp120 by the antibodies [26]. The HPV L1 protein plays a major role in receptor binding to host cells [5] and is the main component of the current HPV prophylactic vaccines. Due to the complex design of the multivalent L1-VLP vaccines, the vaccines cannot prevent all types of HPV infection, and some HPVs that can cause mucosal cancer cannot be covered. Although L2 only induce low titers of antibody, it can produce broadly cross-neutralizing antibodies against heterologous HPV types and might serve as a potential common HPV vaccine antigen [27]. Therefore, assessment on the potential glycosylation sites in L1 and L2 proteins may help us understand the antigenic divergence between different lineages and improve vaccine design. Zhou et al. reported that glycosylated L1 of HPV remained in the endoplasmic reticulum and was not transported for viral particle assembly, suggesting that glycosylated L1 might not be important for virion assembly [28], but the role of L1 glycosylation in receptor or antibody binding remains to be explored. 

A trinucleotide codon is used to encode one standard amino acid, and most amino acids are coded by more than one codon, except Met and Trp. The codons coding for the same amino acid are called synonymous codons. The usage of synonymous codons may vary between and within species, which is called codon usage bias (CUB). The CUB of organisms is largely influenced by natural selection and mutational pressure [29,30,31]. Mutational pressure is determined by the nucleotide composition of the sequence, while natural selection pressure may be affected by translational pressure, gene expression level, protein secondary structure and other factors. The translational pressure comes from the host tRNA pool. As viruses rely on the host translational machinery to synthesize proteins, they may encode codons that best fit the host tRNA pool to increase resource usage. The codon usage patterns of some viruses may be similar to those of the host in order to express viral proteins efficiently [32,33]. However, it has also been found that some viruses may have CUB different from their host to escape from the host’s immune system [34]. It has been shown that the genera *Alphapapillomavirus* and *Betapapillomavirus* have different CUB, which may be related to the histological specificity of the papillomaviruses [35]. CUB was correlated with high A + T content at the 3rd codon position of HPV genes [36]. Optimized codon usage could enhance the expression levels of HPV16 E6 and E7 proteins in mammalian cells, and was suggested for the development of therapeutic vaccines for cervical cancer [37,38]. Understanding the CUB of genes might reveal the potential mechanism underlining persistent HPV16 infection.

The rapid accumulation of HPV16 genome data has provided a new opportunity for extensive and in-depth research on the genetic diversity of HPV16. In this study, we aimed to explore the genomic mutation profiles and the glycosylation site distribution for surface proteins in different HPV16 sublineages. The subsequent findings would help us further understand the heterogeneity between the lineages/sublineages and how such differences might influence surveillance and vaccine application. To further understand the virus–host interaction mechanism of HPV16, we also comprehensively analyzed the codon usage patterns of the eight HPV16 ORFs and compared their CUB with that of humans.

## 2. Materials and Methods

### 2.1. Data Preparation

A total of 3729 complete sequences of HPV16 genomes were retrieved from the National Center for Biotechnology Information (NCBI) (http://www.ncbi.nlm.nih.gov/Genbank/) as of 13 May 2020. In order to obtain high quality genomes, these sequences were processed as follows: (1) sequences with a length of 7000–8500 bp and ambiguous sites less than 5 were kept; (2) sequences that contain 70 or more consecutive “N” (about 1% complete sequence) were removed; (3) sequences were aligned by MAFFT v7.407 (Japan) [39]; (4) the aligned sequences were checked in BioEdit v7.0.5 (Raleigh, USA) [40] and low-quality sequences and those with early stop codons were removed. Finally, a total of 1597 genomes were included for this study. The HPV16 ORFs (E1, E2, E4, E5, E6, E7, L1 and L2) were extracted based on the NCBI record of the HPV16 reference genome (Accession Number K02718), except for E6. To comply with the abundant mutational investigations on E6, the starting position of E6 was set to 104 in the reference genome. The ORF sequences were translated into amino acid sequences to ensure correct reading frames with BioEdit. The detailed information of the genomes, such as host origins, geographical locations and collection time, is provided in Supplementary Table S1. 

### 2.2. Phylogeny Reconstruction and Lineage/Sublineage Classification

Maximum likelihood phylogeny was constructed with IQ-TREE (Austria) using TVM+F+I+G4 nucleotide substitution model with 1000 ultrafast bootstrap implementation [41,42,43]. The nucleotide difference between all sequences and the reference sequences was calculated with R package (New Zealand) seqinr v3.6-1. According to the phylogenetic topology and sequence differences (inter-lineage difference: 1–10%; inter-sublineage difference: 0.5–1%), all sequences were assigned to lineages and sublineages for downstream analysis. The reference sequences of different lineages/sublineages were obtained from GenBank [19], with their accession numbers as follows: K02718 (A1), AF536179 (A2), HQ644236 (A3), AF534061 (A4), AF536180 (B1), HQ644298 (B2), KU053915 (B3), KU053914 (B4), AF472509 (C1), HQ644244 (C2), KU053920 (C3), KU053925 (C4), HQ644257 (D1), AY686579 (D2), AF402678 (D3) and KU053931 (D4).

### 2.3. Mutation Detection of ORFs

Nucleotide sequences of the eight ORFs were compared against the reference genome (K02718) to identify mutations. The amino acid mutations resulting from the nucleotide mutation were also determined.

### 2.4. Identification of Potential Glycosylation Sites in L1 and L2 Proteins

L1 and L2 sequences were translated into protein sequences with BioEdit. The potential glycosylation sites were determined by identification of the N-linked glycosylation motifs (N-X-T/S, X: any amino acid except for P) in the protein sequences.

### 2.5. Nucleotide Composition Analysis

Calculations of the GC content at the 1st, 2nd and 3rd codon positions (GC1, GC2, GC3) and the average content of GC1 and GC2 (GC12) of all ORFs were conducted with R package SADEG v1.0.0 [44]. 

### 2.6. Analysis of Effective Number of Codons

Effective number of codons (ENC) was used to evaluate the overall codon preference of HPV16 genes, which is independent of gene length and amino acid (aa) composition. When only one codon is used for each amino acid, the ENC value will be 20. If all codons are used equally, the value would be 61 [45]. The lower the ENC value, the stronger the bias for codon usage. ENC values were calculated using R package SADEG [44]. The ENC plot (ENC plotted against GC3) [45] could be used to assess if other factors are engaged in shaping the CUB besides mutational pressure. The standard curve in the ENC plot represents the expected ENC values. If the calculated ENC value equals the expected one, the codon usage is only influenced by mutational pressure. Otherwise, selection pressure may be involved. The expected ENC was calculated as below, with S indicating GC3.

(1) $$ENC_{expected}=2+S+\left(\frac{29}{S^{2}+{\left(1-S\right)}^{2}}\right)$$

### 2.7. Neutrality Plot Analysis

Both mutational pressure and natural selection can affect CUB. Nucleotide mutations at the 3rd codon positions usually cause synonymous mutation at the protein level, while those at the 1st and 2nd position tend to cause nonsynonymous mutations, which indicates natural selection. A regression line was drawn by plotting GC12 against GC3 to measure the contribution of mutational and natural selection pressure to CUB. If the regression line is parallel to the diagonal (i.e., slope = 1), mutational pressure is the major factor contributing to CUB. Otherwise, natural selection also plays a role [46].

### 2.8. Codon Usage Frequency Analysis

Relative synonymous codon usage (RSCU) can be used to compare codon usage of genes with different lengths and amino acid compositions. It is assumed that the codons of the same specific amino acid have equal usage, and the ratio of the actual codon usage frequency to the expected frequency is defined as the RSCU value [47]. RSCU values of <0.6, 0.6–1.6, >1.6 indicate low, normal and over usage of the codon [46]. The average RSCU data of humans originated from work by Malik et al. [48], while the mean RSCU values of HPV16 ORFs were calculated by R package SADEG v1.0.0. [44]. 

## 3. Results

### 3.1. Classification of HPV16 Lineages and Sublineages

Using 1597 full genomes (Supplementary Table S1), we constructed a maximum likelihood tree (Supplementary Figure S1) and conducted lineage/sublineage classification based on the criteria proposed by Chen et al. [17]. Only one sequence was not assigned to any lineage/sublineage because of its long distance to the other known lineages. In summary, we obtained 1352 (84.7%) sequences from lineage A, 34 (2.1) from lineage B, 56 (3.5%) from lineage C, and 154 (9.6%) from lineage D (Supplementary Table S2). Of all the sequences in lineage A, 1053 (77.9%) genomes belonged to sublineage A1 (Table 1, Supplementary Table S2), followed by sublineages A2 (204), A4 (84) and A3 (11). Unfortunately, the number of genomes in several B and C sublineages was less than five. Other sublineages with more than 10 sequences included B1 (28), C1 (50), D1 (12), D2 (35), D3 (95) and D4 (12).

### 3.2. Mutations Identified across the HPV16 Genome

**Figure 1:**
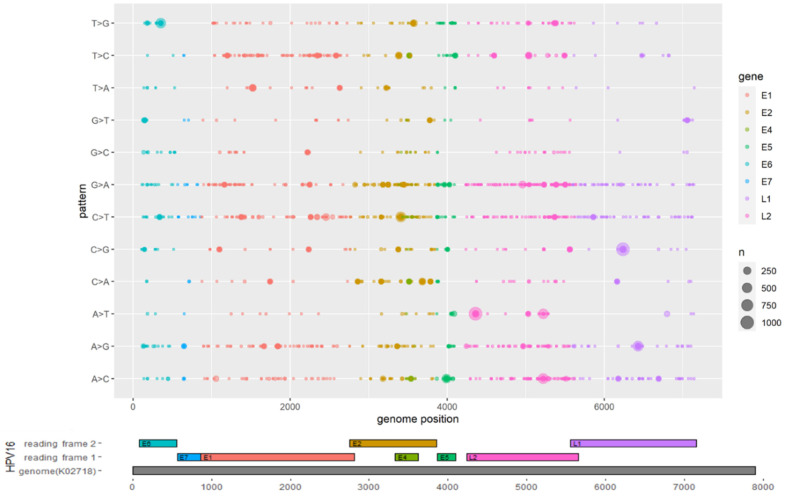
Mutation distribution across the HPV16 genome. The x axis shows HPV16 gene positions, and the y axis shows the 12 nucleotide mutation patterns. The bubble size indicates the occurrence of nucleotide mutations.

As HPV sublineages displayed heterogeneity in geographical distribution and carcinogenic ability, we sought to identify mutations that significantly differ between the lineages and sublineages. Sites in the ORFs that differ from the reference sequence (K02718) were identified as mutation sites. The distributions of mutations by gene are shown in Figure 1. The L2 and E2 ORFs of HPV16 showed higher levels of genomic diversity than other genes, with 6459 and 6894 mutations detected in E2 and L2, respectively. In contrast, E7 was relatively conserved, with only 183 mutations observed (Supplementary Table S3, Figure 1). Interestingly, G-to-A and C-to-T transitional mutations occurred more frequently than the other mutation types, including A-to-G and T-to-C transitional mutations.

To identify sublineage-conserved genetic changes, mutations occurring in over 90% of sequences of the sublineages that contained more than 10 sequences were further identified. There were at least 25 nucleotide sites that displayed fixation in at least one sublineage (Table 1, Supplementary Table S3). Mutations including E2 T3223A, L2 A4967G, L2 A5032T, L2 T5366G and L2 T5384G were uniquely associated with lineage D, while E5 A4054T, E5 G3881A, and L2 A5288G were uniquely associated with lineage B or sublineage B1, and E6 G132T and L2 A5288C were associated with sublineage C or sublineage C1. Several other mutations were found to be sublineage-specific, including E1 C1415T for C1, E2 G3412A for D1, E2 G3415A for D2, E2 T3386C and L1 A6801T for D3, and E2 C3158G for D4. The HPV16 E6 T350G (L83V) mutation, which was strongly associated with cervical cancer progression [49,50], was highly conserved in lineage D, but was also observed in sublineages A1 and A2. The conserved mutations may be useful for the lineage or sublineage identification based on nucleotide polymorphism.

### 3.3. Glycosylation Analysis of HPV16 L1 and L2 Proteins

**Figure 2:**
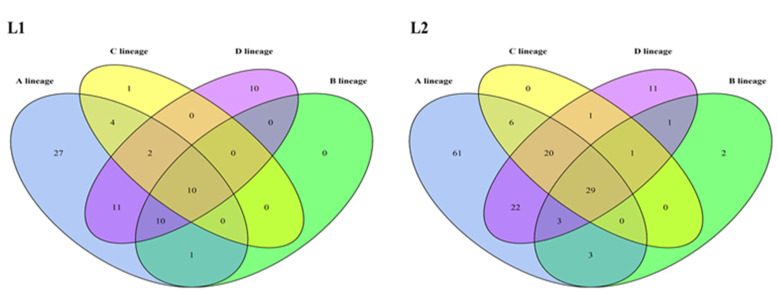
The lineage distribution of potential glycosylation sites in L1 and L2 proteins.

To explore the variations of HPV16 L1 and L2 proteins, the amino acid sequences of L1 and L2 of 1597 HPV16 genomes were predicted for glycosylation sites. The A1 sublineage had the largest number of potential glycosylation sites in L1 and L2 proteins, which may be due to the abundant sequences within this sublineage (Supplementary Tables S4 and S5). Ten and twenty-nine glycosylation sites were identified in all lineages for L1 and L2 proteins, respectively (Figure 2). Some glycosylation sites were lineage-specific. In the L1 protein, 27 glycosylation sites were observed only in lineage A, 1 in lineage C and 10 in lineage D. In the L2 protein, 61 glycosylation sites were only found in lineage A, 2 in lineage B and 11 in lineage D. Collectively, the L1 and L2 glycosylation sites of lineage D displayed the largest differences from those of the HPV16 prototype lineage, i.e., lineage A. These lineage-specific glycosylation sites may play an important role in host cell recognition and the immune escape process. 

### 3.4. Nucleotide Composition of HPV16 Genomes

Our analysis on nucleotide contents showed that HPV16 genomes are AT-rich (Supplementary Table S6). The mean nucleotide content of A and T for the eight ORFs (E1, E2, E4, E5, E6, E7, L1 and L2) was 31.83% and 28.95%, respectively, higher than that of C and G. The mean G+C% of the eight ORFs ranged from 33.46% (E5) to 50.13% (E4). Comparison by codon positions showed that the third codon positions contained low GC content (15.07–41.85%), with E1 (18.62%) and L2 (15.07%) showing extremely low values. These indicated that the third codon position mainly accounted for the nucleotide composition bias of HPV16.

### 3.5. The Effect of Mutational and Natural Selection Pressure on CUB of HPV16

**Figure 3:**
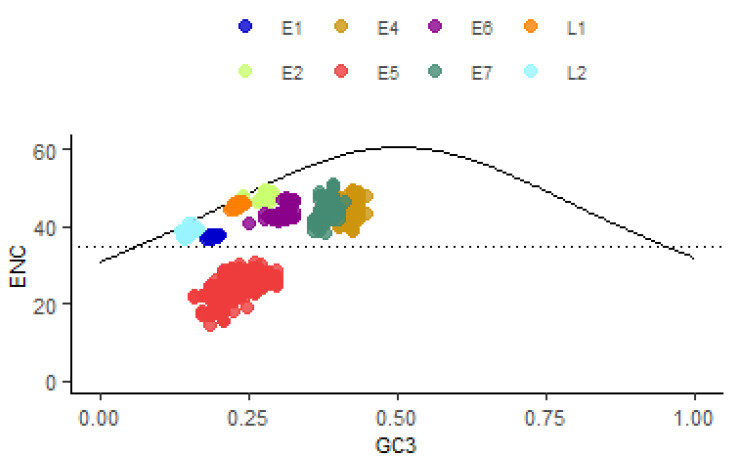
ENC plot of the eight ORFs of HPV16. The continuous curve plots the relationship between GC3 and ENC in the absence of selection. The horizontal dotted line represents the ENC value of 35. Almost all points lie below the curve.

The ENC plot is used to find out if factors other than mutational pressure are affecting CUB. In Figure 3, the curve represents the expected ENC determined by GC3, and the points represent the actual ENC values of the eight ORFs. The strains of the different HPV16 lineages had similar ENC values. Almost all ENC values of HPV16 ORFs lie below the standard curve, suggesting that natural selection also influences the codon usage pattern of HPV16. The mean ENC value for the HPV16 ORFs was 41.27, and seven out of the eight ORFs had an ENC larger than 35, indicating that the overall extent of CUB in HPV16 genomes was low. Interestingly, E4, E5 and E7 exhibited relatively lower ENC than expected, especially the E5 ORF (the mean ENC value was 24.95), implicating relatively high CUB. Although ENC is generally independent of gene length, these may still be influenced by the extremely short length of the three ORFs, which are less than 100aa (E4, 95aa; E5, 78aa; E7, 98aa).

**Figure 4:**
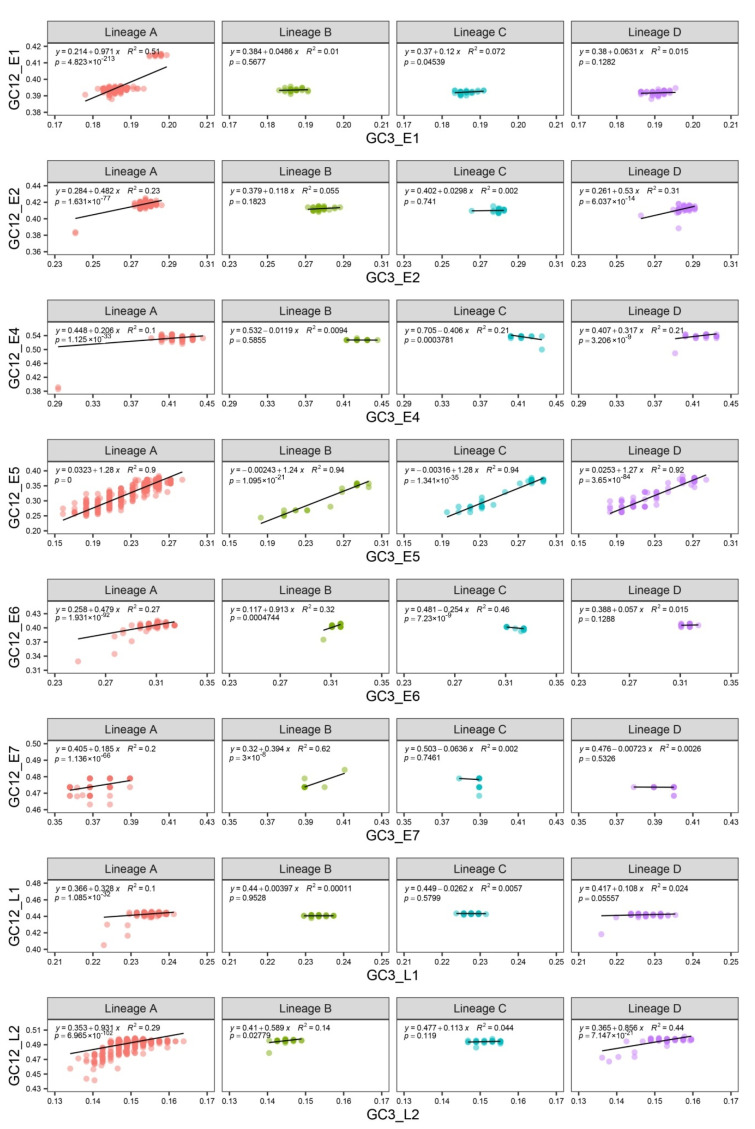
Neutrality plot analysis of GC12 and GC3 for HPV16 ORFs.

To further understand the influential extent of mutational pressure and natural selection in HPV16 CUB, regression analysis was conducted using GC12 (the mean GC content at the first and second codon positions) and GC3 (GC content of the third codon position) of each ORF (Figure 4). Neutrality plots of the ORFs were conducted for each lineage to reveal their differences. For lineage A, we observed a high correlation between GC12 and GC3 for the eight ORFs. The regression slopes for E1, E2, E4, E5, E6, E7, L1 and L2 were 0.971, 0.482, 0.206, 1.28, 0.479, 0.185, 0.328 and 0.931, respectively. Therefore, the contribution of natural selection to the CUB of the above ORFs was 2.9%, 51.8%, 79.4%, 28%, 52.1%, 81.5%, 67.2% and 6.9%, respectively. For lineages B, C and D, most of the correlation results were hard to interpret because of the large *p* values (>0.1) or small *R*^2^ (<0.1). Nevertheless, natural selection was estimated to account for 24%, 8.7%, 60.6% and 41.1% of the CUB for E5, E6, E7 and L2 in lineage B; 59.4%, 28% and 74.6% of the CUB for E4, E5, and E6 in lineage C; and 47%, 68.3%, 27% and 14.4% of the CUB for E2, E4, E5 and L2 in lineage D. In summary, natural selection seems to play a major role in shaping the CUB of HPV16 genes, except for E1, E5 and L2.

### 3.6. Analysis of RSCU

**Figure 5:**
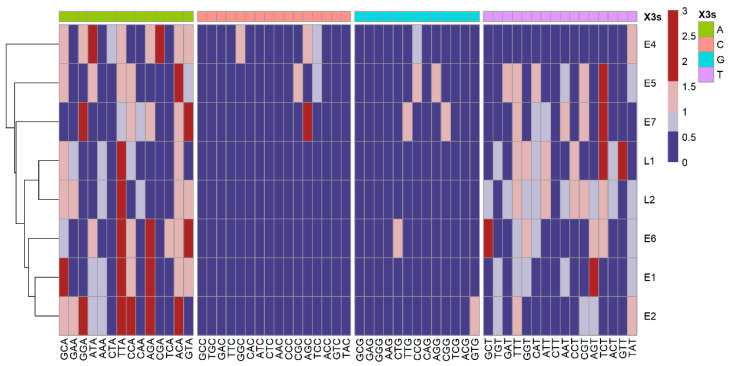
Relative synonymous codon usage (RSCU) analysis revealed over-representation of codons ending in A/T in HPV16 ORFs. Columns correspond to the 59 codons (three stop codons and those for Trp, Met were excluded). Rows correspond to the eight ORFs. Blue cells indicate under-represented codons (RSCU < 0.6) and red cells indicate over-represented codons (RSCU > 1.6). “X3s” indicates the nucleotide at the 3rd codon position.

To measure the usage variations of each codon, we calculated the RSCU values for HPV16 ORFs (Figure 5). As the RSCU results were similar among the four lineages, we only showed the integrated results for the whole dataset. The RSCU of most codons ending in G/C was below 0.6, indicating that the usage frequency of these codons was relatively low. In contrast, RSCU values greater than 1.6 were mostly found in codons ending in A/T, indicating high usage preference. The top highly used codons included GCA for alanine, CCA for proline, ACA for threonine, TTA for leucine, and AGA for arginine. TTA (leucine) was highly used in both L1 and L2 genes, AGA was the highly used codon in the E6 gene, while the E7 gene mostly preferred the codon of GTA (Supplementary Table S7). This finding was consistent with the high AT content in the nucleotide composition of the ORFs. 

**Figure 6:**
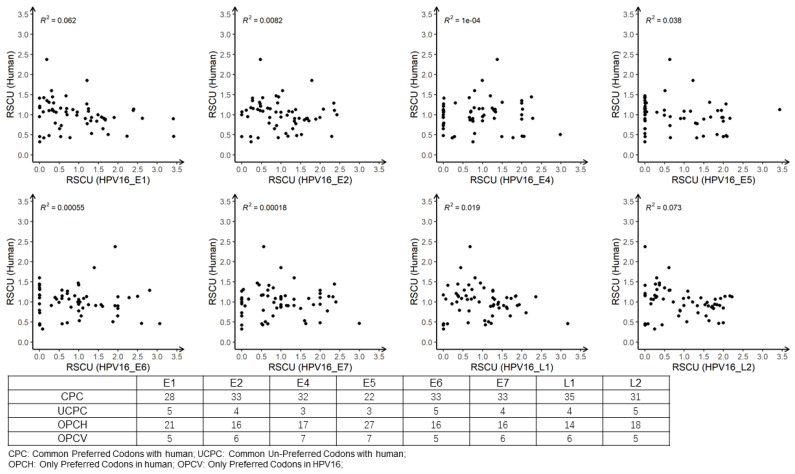
Pairwise correlation analysis of RSCU for 59 codons in eight HPV16 ORFs versus those of humans. The R-squared values of linear regression analysis are shown. The embedded table denotes the number of commonly preferred (RSCU ≥ 0.6) codons and unpreferred (RSCU < 0.6) codons for HPV16 and human genes, and the number of preferred codons in humans but unpreferred in HPV16 and preferred codons in HPV16 but unpreferred in humans.

To understand the codon usage compatibility between virus and host, a correlation analysis between RCSU values of the eight HPV16 ORFs and those of humans was performed (Figure 6). The low R square values indicated that the codon usage preferences of the two species only partially overlapped, with around 22–35 commonly preferred codons (i.e., normal and over usage) and 3–5 commonly unpreferred codons (Figure 6, bottom panel). This left 14–27 codons that were only preferred by humans and 5–7 codons only preferred by HPV16. These results suggested that HPV16 was adapted in using the host translational machinery, but also avoided over competition with cellular protein production to reduce stimulation of the host immune response, which would help its persistence in humans.

## 4. Discussion

Mutations in viral genes are important for variant identification and functional annotation. In our results, the most common mutations were T350G in the E6 gene and A647G in the E7 gene (Table 1). It was reported that these two mutations were related to the development of cervical cancer [51,52,53] and E7 A647G may be more common in China [54]. Our mutation analysis showed that T350G mutation was detected in all viruses of lineage D and some strains of A/B lineages, while E7 A647G was observed in almost all A4 and C1 sublineages. Another mutation, HPV16 E6 D25E, which was associated with an elevated risk for the development of invasive cervical cancer [55], was not identified as a conserved mutation in our research. Variations in E6 (E/G131T) may alter the HLA-B7 peptide binding epitope to help HPV16 escape from immune surveillance [56]. Previous research reported that HPV16 sublineages could be classified based on 13 and 32 phylogenetically distinguishing positions in E6 and the URR [20]. In this study, 35 lineage/sublineage-conserved mutations were identified. These mutations may help determine the HPV16 lineages/sublineages in epidemiological studies of HPV16. We also identified high levels of G-to-A and C-to-T mutations, which may have resulted from the deamination effects of the APOBEC or AID protein families [57], especially APOBEC3A [58]. Such mutations may occur when single-stranded DNA is exposed during the transcriptional process, and the unusually high mutation spectrum may facilitate the emergence of tumors [59].

Glycosylation modification of viral surface proteins is critical for viral infectivity and antigenicity, as documented for influenza viruses [60], dengue viruses [61] and HIV [26], which is a factor to be considered during vaccine application. Among the four HPV16 lineages, lineage D contained the largest number of different glycosylation sites in L1 and L2 proteins from lineage A (Figure 2). Godi et al. showed that compared with HPV16 lineage A, lineages B, C, and D exhibited slightly (<2-fold) reduced sensitivity to nonavalent vaccine sera [62]. The unique glycosylation sites existing on the L1 proteins of lineages B, C and D, especially D, might be one of the determinants accounting for this difference. Additional studies are needed to demonstrate the function of glycosylation sites of HPV16 L1 and L2 proteins and the impact of glycosylation on the design of HPV vaccines.

Our nucleotide composition analysis showed that the A+T content of HPV16 was higher than the G+C content in most HPV16 ORFs. Zhao et al. [36] analyzed 79 HPV types and showed that the E4 gene was GC-rich while the other open reading frames were AT-rich, which was similar to our findings. It has been shown that GC3 was associated with the CUB of the organism [29,63,64], GC-rich codons were more likely to end in GC, and vice versa. We found that the GC3 content of the HPV ORFs ranged from 15.07% to 41.85%, reflecting preference to A/T-ending codons. Consistently, we found that the RSCUs were higher for codons ending in A/T. In our analysis, the ENC values of the HPV16 genes were above 35, except that of E5 gene, indicating a low CUB and possibly low gene expression level [63,65]. The statement that ENC calculation was generally independent of gene length was true for genes with over 100 codons but may not be applicable for short genes [45]. Therefore, the ENC results for the three ORFs (E4, E5 and E7) with less than 100 codons should not be over-interpreted. Our neutrality analysis indicates that natural selection was the main factor affecting the CUB of HPV16 E2, E4, E6, E7 and L1, while mutational pressure was the major force affecting the CUB of E1, E5 and L2. We suspected that genes (E2, E6, E7 and L1) encoding proteins with more frequent interactions with the host cellular factors and higher immune stimulating potential may face heavier natural selection pressure. E4 is located within the E2 ORF, and its CUB may be affected by that of E2. While both L1 and L2 are capsid proteins, L1 is the major component exposed in the surface to interact with the immune system [12]. We also found that the codon usage of HPV16 did not fully overlap with that of humans, which might help the virus better evade host immunity to facilitate persistent infection in humans. 

Using a large amount HPV16 complete genomes, we have comprehensively investigated the mutation profiles across the HPV16 genes, potential glycosylation site distribution in surface proteins and the codon usage patterns of all eight ORFs. These findings might provide important implications for variant identification and novel vaccine development, and give hints on the virus–host interaction mechanism supporting chronic viral infection in humans. Currently, the available HPV16 genomes are mainly from lineage A, especially sublineage A1. Increased genomic surveillance around the world may further reveal the complete sublineage diversity of HPV16 and improve the genomic research on these viruses. 

## Figures and Tables

**Table 1:** Mutation profiles of HPV16 sublineages.

ORF	Nucleotide Mutation	Amino Acid Mutation	Proportion of Sequences with the Corresponding Mutations in Each Sublineage (%)									
			A1	A2	A3	A4	B1	C1	D1	D2	D3	D4
			(*n* = 1053)	(*n* = 204)	(*n* = 11)	(*n* = 84)	(*n* = 28) (B, *n* = 34)	(*n* = 50) (C, *n* = 56)	(*n* = 12)	(*n* = 35)	(*n* = 95)	(*n* = 12)
E1	T1220C	V119A							100		1.1	
	C1415T	T184I						100 (89)				
	C1598T	P245L					92.9 (76)				1.1	
	A1667G	H268R							100	100	100	100
	T2252C	F463S	0.1						91.7			
	T2253C	F463S							91.7			
	T2342C	F493S	0.1			1.2				100	95.8	
	C2343T	F493S								100	94.7	
	T2354C	L497P	0.5					100 (100)				
	T2375C	L504P	0.3	2.5			3.6 (2.9)			100		
	C2456T	T531I	0.1	99.0								
E2	C3158G	T135R										100
	A3180C	E142D					14.3 (14.7)	6 (7.1)			97.9	
	T3223A	L157I/M ^a^							100	100	100	100
	T3383C	I210T	1.7	100		100						
	T3386C	I211T									95.8	
	G3412A	A220T							100			
	G3415A	A221T								100		
	G3430A	A226T						100 (98.2)		2.9		
E5	G3881A	A7T					100 (100)					
	A4054T	L64S/F ^b^					100 (100)	2 (1.8)				
	A4089T	H76L				97.6						
E6	G132T	R10I						98 (87.5)				
	C143G	Q14D					92.9 (98.2)	98 (99.5)				
	T350G	L83V	47.8	21.6			3.6 (14.7)		100	100	100	100
E7	A647G	N29S				98.8		100 (89.3)				
L1	A6178C	N207T				41.7	14.3 (11.8)	78 (75)	8.3	5.7	100	
	T6480C	S308P					3.6 (2.9)	100 (100)				
	A6801T	T415S									97.9	
L2	A4967G	T245A	0.1						100	97.1	98.9	100
	A5032T	L266F							100	100	100	100
	A5288C	T353P						100 (89.3)				
	A5288G	T353A					100 (97.1)					
	T5366G	S379A/V ^c^							100	97.1	96.8	100
	T5384G	S385A							100	97.1	100	100

Note: mutation sites were determined for sublineages with more than 10 sequences, and only those mutations occurring in >90% of the sequences in a certain sublineage were shown. Blank space indicates that there were few/no corresponding mutations in the sublineage or that sublineage contained less than 10 sequences. As multiple sublineages of B and C lineages contained less than 10 strains, the overall mutation frequencies were also calculated for B and C lineages were also calculated. The numbers in parentheses indicate the proportion of the mutation in B or C lineage. ^a^ L157I/M: T3223A -> L157I; T3223A and A3224G -> L157M. ^b^ L64S/F: A4054T -> L64F; A4054T and T4053C -> L64S. ^c^ S379V/A: T5366G -> S379A; T5366G and C5367T -> S379V.

## Data Availability

Data supporting the analysis of this study was provided as supplementary material.

## Supplementary Materials

The following are available online at https://www.mdpi.com/article/10.3390/v13071281/s1, Table S1: The detailed information of HPV16 genomes downloaded from public database. Table S2: Summary of the lineage/sublineage distribution of HPV16 genomes. Table S3: All mutations observed in HPV16 ORFs. Table S4: Potential glycosylation sites in L1 proteins of different HPV16 sublineages. Table S5: Potential glycosylation sites in L2 proteins of different HPV16 sublineages. Table S6: The nucleotide composition of the eight ORFs of HPV16 (%). Table S7: The RSCU values of 59 synonymous codons in eight HPV16 ORFs. Figure S1: Phylogeny of HPV16 complete genomes. Maximum likelihood phylogeny was constructed with IQ-TREE using TVM+F+I+G4 nucleotide substitution model. The tree scale is displayed at the bottom.
