# Supplementary material for: Mutation Profiles, Glycosylation Site Distribution and Codon Usage Bias of Human Papillomavirus Type 16
Source: Viruses. 2021 Jun 30;13(7):1281. doi: 10.3390/v13071281 (PMC8310365; doi:10.3390/v13071281)
Supplement: Supplementary file 1 [file viruses-13-01281-s001.zip › viruses-1153003-supplement.pdf]

viruses

Supplementary Materials: Mutation Profiles, Glycosylation Site Distribution and Codon Usage Bias of Human Papillomavirus Type 16

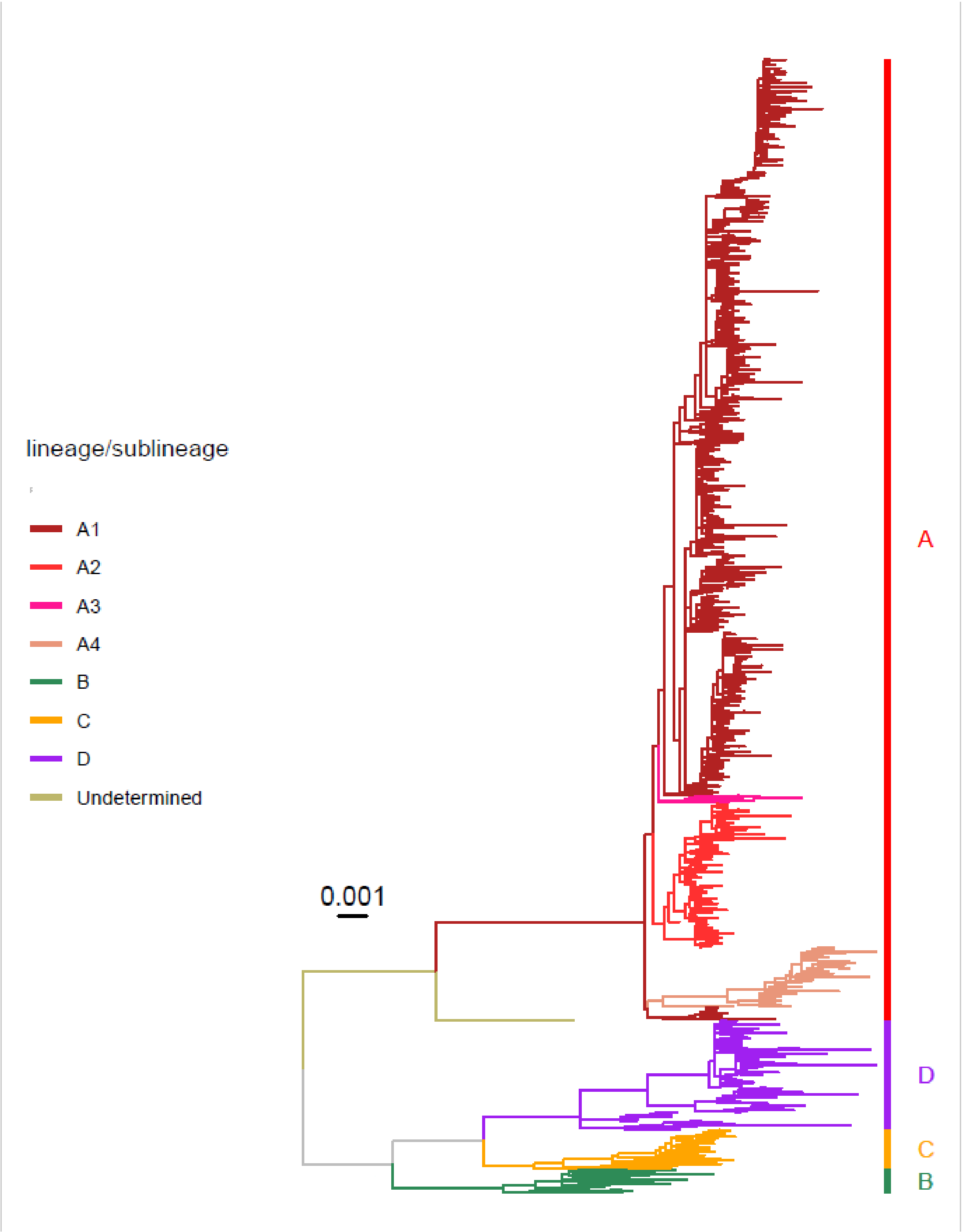

Figure S1. Phylogeny of HPV16 complete genomes. Maximum likelihood phylogeny was constructed with IQ-TREE using TVM+F+I+G4 nucleotide substitution model. The tree scale is displayed at the bottom.
